# A Brave Surgeon

**Published:** 1875-01

**Authors:** 


A Brave Man.—In the transactions of the Minnesota State Medical Society for the present year, Alex. J. Stone, M.D., of St. Paul, makes a report on ovariotomy, describing five cases operated on by himself, *all fatal.* The bravery and honesty of a medical man who can thus afford to publish his failures for the benefit of others, are beyond all cavil.—*Pacific Medical and Surg. Jour.*

